# Olfactory mucosa mesenchymal stem cell‐derived exosomes protect against neuroinflammation after subarachnoid hemorrhage by activating mitophagy

**DOI:** 10.1002/kjm2.12951

**Published:** 2025-02-19

**Authors:** Jian Wang, Jun Peng, Ling Gao, Jun He, Long Lin, Jia‐Meng Li, Ying Xia

**Affiliations:** ^1^ Department of Neurosurgery Haikou Affiliated Hospital of Central South University Xiangya School of Medicine Haikou Hainan Province People's Republic of China

**Keywords:** exosomes, mitophagy, neuroinflammation, olfactory mucosa mesenchymal stem cells, subarachnoid hemorrhage

## Abstract

Subarachnoid hemorrhage (SAH) can lead to significant acute neuroinflammation, with treatment outcomes often being inadequate. Olfactory mucosa mesenchymal stem cells (OM‐MSCs) have promising therapeutic potential in nerve regeneration and functional recovery. This investigation sought to elucidate the functional mechanisms through which exosomes derived from OM‐MSCs provide protection against neuroinflammation following SAH. Mouse OM‐MSCs and their exosomes were isolated and characterized using various techniques, including transmission electron microscopy, immunofluorescence staining, Western blotting, flow cytometry, and nanoparticle tracking analysis. Hemin‐induced HT22 cells were subsequently utilized to assess the impact of OM‐MSC‐derived exosomes on the inflammatory response, apoptosis, and mitophagy through ELISAs, Western blotting, qPCR, flow cytometry, and immunofluorescence staining. The impacts of exosomes on neuroinflammation and neuronal damage in SAH model mice were assessed using qPCR, ELISAs, Western blotting, immunofluorescence staining, and TUNEL staining. Exosomes derived from OM‐MSCs had the capacity to reduce the levels of proinflammatory factors (IL‐6, IL‐1*β*, and TNF‐*α*) and promote apoptosis in hemin‐induced HT22 cells. Exosomes alleviated neuroinflammation and neuronal injury post‐SAH, as evidenced by the increase in modified Garcia scores, reduction in the brain water content, decrease in blood–brain barrier permeability, decreases in inflammatory marker levels, and reduction in apoptosis rates. Notably, the protective effects of exosomes derived from OM‐MSCs on neuroinflammation and apoptosis, both in vitro and in vivo, were mediated via the activation of mitophagy. These findings provide a fresh perspective for subsequent clinical research in the domain of prevention and treatment strategies.

## 1 INTRODUCTION

Subarachnoid hemorrhage (SAH) is a specific type of stroke that occurs when a blood vessel in the brain ruptures, causing blood to leak into the subarachnoid space and leading to symptoms such as severe headache, nausea, vomiting, and altered consciousness. SAH accounts for 5%–10% of all strokes,^1^ with the primary cause being a ruptured intracranial aneurysm.^2^ After SAH, brain injury can be categorized into early brain injury (EBI; within 72 h) and secondary brain injury (after 72 h). Research indicates that EBI is linked to the development of delayed cerebral ischemia,^3^ whereas secondary brain injury is related to a poor prognosis for SAH patients.^4^ Approximately 30%–50% of SAH survivors experience delayed neurological issues,^5^ which impacts their quality of life and creates a greater socioeconomic burden.^6^ Therefore, investigating the mechanisms of nervous system damage caused by SAH is crucial for obtaining a clinical understanding.

EBI is the primary factor contributing to negative outcomes following SAH, resulting in pathological alterations such as the disruption of the blood–brain barrier, brain edema, and neuronal apoptosis post‐SAH.^6^, ^7^, ^8^ The pathophysiological processes that occur after SAH are intricate and include neuroinflammation, oxidative stress, microcirculatory dysfunction, apoptosis, and autophagy.^9^ Following hemorrhage, the release of hemoglobin and its breakdown products, organelle dysfunction, and the activation and infiltration of inflammatory cells leads to increased expression of oxidative and inflammatory factors. These factors subsequently result in intracranial hypoperfusion pressure, an impaired blood–brain barrier, brain edema, neuronal apoptosis, and other neural injuries.^10^ Neuroinflammation is a pivotal pathological mechanism of EBI triggered by SAH.^11^ Research indicates that inhibiting neuroinflammation can mitigate brain edema and neuronal damage in rat models post‐SAH, ameliorating EBI and ultimately improving the prognosis of SAH.^12^ Therefore, interventions targeting neuroinflammation in individuals with SAH can mitigate EBI and contribute to enhanced neurological outcomes.

Mesenchymal stem cells (MSCs) have been utilized in the management of nerve function impairment following cerebral hemorrhage.^13^ Additionally, MSCs have been extensively employed in both experimental and clinical investigations, demonstrating their therapeutic potential in SAH.^14^ Studies have indicated that bone marrow MSC therapy can mitigate the neurobehavioral impairments and inflammatory responses associated with EBI post‐SAH. The use of bone marrow MSC exosomes has been shown to ameliorate EBI after SAH because of the antiapoptotic and anti‐inflammatory effects of these exosomes.^15^ Furthermore, the neuroprotective effect of exosomes derived from umbilical cord MSCs on neuronal damage subsequent to intraventricular hemorrhage may be attributed to the modulation of inflammatory responses, suggesting that umbilical cord MSC exosomes possess neuroprotective properties that can suppress inflammation and neuronal cell death.^16^ Olfactory mucosa MSCs (OM‐MSCs), a type of stem cell with versatile differentiation capabilities, offer advantages such as high safety, ease of procurement, and an absence of ethical concerns. OM‐MSCs have been shown to facilitate nerve regeneration and functional recovery.^17^ During neurogenesis, exosomes derived from OM‐MSCs have been found to suppress neuroinflammation,^18^ although their specific regulatory effects on neuroinflammation in individuals with SAH have yet to be fully elucidated.

Numerous studies suggest that the process of neuroinflammation may be suppressed through the stimulation of mitophagy.^19^ Melatonin‐induced mitophagy exerts a protective effect on EBI subsequent to SAH by hindering NLRP3 inflammasome activation.^20^ Nevertheless, the specific impact of exosomes derived from OM‐MSCs on neuroinflammation through the activation of mitophagy remains unexplored. Our investigation aimed to clarify the underlying mechanism by which exosomes from OM‐MSCs alleviate neuroinflammation following SAH by promoting mitophagy.

## 2 METHODS

### 2.1 Ethics statements and experimental animals

All in vivo experiments conducted in this study were approved by the Institutional Ethics Committee. Male C57BL/6 mice (weighing between 25 and 30 g) were purchased from Shanghai SLAC Laboratory Animal Company (China). These mice were randomly divided into relevant groups (Experiment 1 and Experiment 2), with 6 mice in each group. The mice were housed in a controlled environment (temperature: 22 ± 1°C, humidity level: 60 ± 5%) with a 12‐h light–dark cycle. They had unrestricted access to food and water.

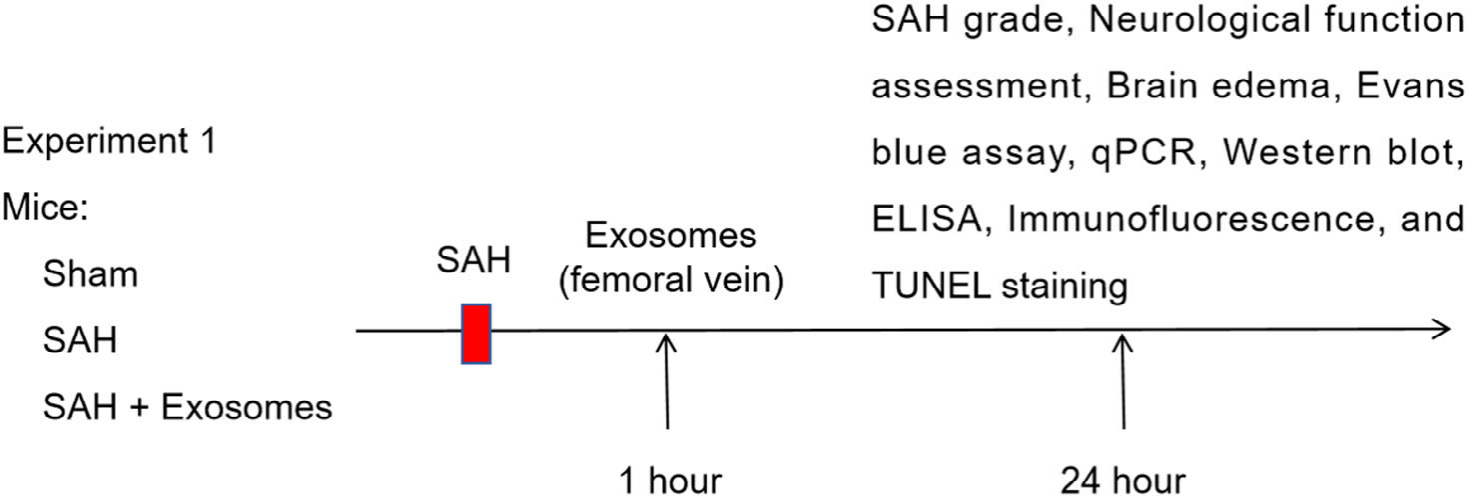

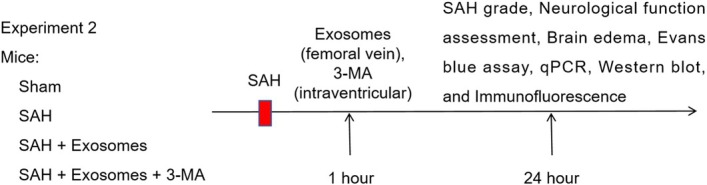

### 2.2 Isolation and identification of OM‐MSCs

OM‐MSCs were isolated and cultured according to a previously published protocol.^21^ After anesthesia with 20 μL/g pentobarbital sodium (0.3%) was administered, the mice were decapitated. The skin, lower jaw, and bone covering the nasal cavity were subsequently removed with sterile surgical instruments to avoid any microbial contamination. Forceps were employed to separate the olfactory mucosa from the septum meticulously under a dissecting microscope to ensure the integrity of the tissue sample. The olfactory mucosa obtained from the mice was subsequently transferred promptly to prechilled DMEM/F12 (GIBCO, USA), which was supplemented with a cocktail of antibiotics (100 U/mL penicillin and 100 μg/mL streptomycin) to prevent possible bacterial and fungal growth during the initial handling stage. Next, the olfactory epithelium was carefully removed by gentle mechanical agitation and aspiration, leaving behind the lamina propria. The remaining lamina propria was shredded into small fragments of approximately 1–2 mm^3^ using sterile scissors and transferred to medium containing collagenase II (0.5%) and 0.01% DNase I for 1 h of cultivation at 37°C in a humidified incubator with 5% CO₂. The addition of DNase I helps degrade the extracellular DNA released during tissue dissociation, preventing cell clumping and increasing the cell yield. After enzymatic digestion, the cell suspension was centrifuged at 300 × *g* for 10 min to pellet the cells. The supernatant was discarded, and the cells were resuspended in fresh, complete DMEM/F12 supplemented with 10% fetal bovine serum (FBS), 2 mM L‐glutamine, 100 U/mL penicillin, and 100 μg/mL streptomycin. The morphology of the OM‐MSCs that had been isolated and cultured to passage 3 was examined using a microscope. The levels of Vimentin (10366‐1‐AP, 1:100, Proteintech, Wuhan, China) and Nestin (19483‐1‐AP, 1:100, Proteintech) in these cells were measured using an immunofluorescence assay. The surface markers of OM‐MSCs, including CD34 (ab81289, 1:50, Abcam, Cambridge, MA, USA), CD45 (ab10558, 10^6^ cells using 1 μg, Abcam), CD73 (ab317462, 1:50, Abcam), and CD44 (ab243894, 10^6^ cells using 1 μL, Abcam), were detected by flow cytometry.

### 2.3 Collection of OM‐MSC exosomes

Exosomes were collected from the conditioned medium of OM‐MSCs via ultracentrifugation, as previously described.^22^ Briefly, the OM‐MSCs cultured to 90% confluence were incubated for 48 h in DMEM/F12 supplemented with exosome‐depleted serum (10%) and sequentially centrifuged at 4°C (300 × *g* for 10 min; 2000 × *g* for 10 min; and 10,000 × *g* for 30 min) to obtain the conditioned medium. After filtering with a syringe filter (0.22 μm; Millipore, Billerica, MA, USA), the supernatant was centrifuged (100,000 × *g*) at 4°C for 70 min. After washes with PBS, the suspension was centrifuged (100,000 × *g*, 70 min) again, and the isolated exosomes were resuspended in PBS (100 μL). The morphological observation of the exosomes was subsequently performed using a transmission electron microscope (TEM). A nanoparticle tracking analyzer (NTA) was used to analyze the exosomal particle size distribution. Calnexin (10427‐2‐AP, 1:10000, Proteintech), TSG101 (28283‐1‐AP, 1:5000, Proteintech), and CD81 (27855‐1‐AP, 1:1000, Proteintech) levels were verified by Western blotting.

### 2.4 TEM characterization

For the TEM analysis, a 10 μL aliquot of the exosome suspension was placed on a carbon‐coated copper grid and allowed to adsorb for 5–10 min at room temperature. The excess liquid was subsequently carefully removed with filter paper, and the grid was negatively stained with a 2% uranyl acetate solution for 1–2 min. After air drying, the grid was inserted into a TEM (HT‐7700, Hitachi, Japan) operating at an accelerating voltage of 80 kV. Images were captured at multiple magnifications to clearly visualize the morphology of the exosomes. This staining and imaging process enabled the visualization of the exosome structure, which typically appears as cup‐shaped or spherical vesicles with a distinct membrane bilayer.

### 2.5 NTA characterization

NTA was performed using a NanoSight NS300 instrument (Malvern Panalytical, UK) equipped with a 405 nm laser and a scientific complementary metal‐oxide‐semiconductor (sCMOS) camera. Before the analysis, the isolated exosome sample was diluted to an appropriate concentration with PBS to ensure accurate particle counts and size distribution measurements. A 1 mL aliquot of the diluted sample was loaded into the sample chamber of the NanoSight instrument, and the instrument was set to record videos for 60 s at a frame rate of 30 frames per second. The NTA software (version 3.40) then tracked the Brownian motion of individual exosome particles in the recorded videos and calculated their hydrodynamic diameters based on the Stokes–Einstein equation. This method enables the determination of the size distribution profile of the exosome population, providing quantitative data on the most prevalent particle sizes.

### 2.6 Cell treatments

Mouse hippocampal neurons (HT22) were obtained from Procell (CL‐0697, Wuhan, China) and were grown in DMEM (GIBCO) supplemented with 1% penicillin–streptomycin and 10% FBS (Procell) in a 37°C, 5% CO_2_ incubator. HT22 cells were exposed to hemin (160 μM; H140872, Aladdin, Shanghai, China) for 24 h to induce SAH injury in vitro.^6^ For the treatment process, the cells were administered exosomes derived from OM‐MSCs (40 μg) and/or 3‐methyladenine (3‐MA; 10 mM, M424419, Aladdin).

### 2.7 Exosome uptake

The exosome suspension (100 μL) was combined with 1 mL of PKH26 dye (PKH26GL, Sigma‐Aldrich, St. Louis, MO, USA). Subsequently, HT22 cells (2 × 10^5^ cells per well) were cultivated in 6‐well plates and exposed to PKH26‐labeled exosomes for 6, 12, or 24 h, followed by staining with DAPI. The uptake of exosomes by HT22 cells was detected using a fluorescence microscope (Leica, Wetzlar, Germany).

### 2.8 Cell counting Kit‐8 (CCK‐8)

Upon stimulation with hemin (10–320 μM) for 24 h, HT22 cells (1 × 10^3^ cells per well) were inoculated into 96‐well plates containing 100 μL of medium along with 10 μL of CCK‐8 reagent (CA1210, Solarbio, Beijing, China) and incubated for 2 h. Subsequently, the absorbance (OD) at 450 nm was measured with a microplate reader (Thermo Fisher Scientific, Waltham, MA, USA).

### 2.9 SAH modeling

SAH model mice were induced through endovascular perforation.^23^ Briefly, 5% isoflurane was utilized to anesthetize the mice, and 2% isoflurane was used to sustain anesthesia. The mice were positioned supine, and a surgical incision was made in the central neck region to dissect the common carotid, external carotid, and internal carotid arteries. A 4–0 monofilament was subsequently advanced from the left external carotid artery into the internal carotid artery until resistance was encountered for vessel puncture. Thereafter, the filament was extracted promptly, the external carotid artery was ligated, and the wound was sutured closed. The mice in the sham group underwent a comparable surgical procedure but without the induction of perforation. For the exosome treatment, 400 μg of OM‐MSC‐derived exosomes (in 200 μL of PBS) were infused into the femoral vein of the mice 1 h after SAH. For mitophagy inhibition, 3‐MA (15 mg/kg) was injected intraventricularly into SAH mice. Twenty‐four hours after SAH induction, the mice were sacrificed, and brain tissues were collected for qPCR, Western blots, ELISA, immunofluorescence, and TUNEL staining. A total of 40 mice were modeled in this study, and 5 died within 24 hours after modeling, resulting in a mortality rate of 12.50%.

### 2.10 SAH grade

The SAH grade was evaluated in a blinded manner 24 h after the model was established. In summary, the basal cistern was divided into six distinct regions, and each region was assigned a numerical score ranging from 0 to 3 based on the presence and extent of blood within the subarachnoid space (0 indicating no blood, 1 indicating a small amount, 2 indicating a moderately visible blood clot, and 3 indicating complete arterial blockage by the clot). Mice with an SAH score equal to or less than 8 were excluded.

### 2.11 Neurological function assessment

Neurological performance was evaluated 24 h after SAH induction. In accordance with the modified Garcia scoring system,^24^ six aspects, namely, spontaneous activity (in a cage for 5 min), symmetry of limb climbing, movements of forelimbs (outstretching while held by the tail), climbing the wall of a wire cage, reaction to touch on both sides of the trunk, and response to vibrissae touch, were used to evaluate the neurological function of the mice in each group. The range of scores assessed in the study was from 3 to 3–18 points, with lower scores indicating more severe damage to neurological function.

### 2.12 Brain water content

Brain tissue was collected at 24 h after SAH induction, and the brain water content was utilized to assess brain edema in the mice by detecting the wet and dry weights of the brain tissues (left and right hemispheres). The brain water content was calculated as (wet weight–dry weight)/wet weight × 100%. The brain tissue was placed in an oven at a constant temperature of 100°C for 24 h until a stable weight was achieved, after which the dry weight was measured using an electronic balance.

### 2.13 Blood–brain barrier injury

Twenty‐four hours after SAH, the permeability of the blood–brain barrier was evaluated by injecting 2% Evans blue dye (E104208, Aladdin) into the left ventricle at a dose of 5 mL/kg (1 h before sacrificing the mice). The brains were subsequently separated into left and right cerebral hemispheres and homogenized in trichloroacetic acid (50%). After centrifugation, the supernatant was collected and incubated with ethanol and trichloroacetic acid at 4°C overnight. The concentration of Evans blue was subsequently determined by measuring the absorbance at 630 nm. A more substantial exudation of Evans blue indicates more severe impairment of the blood–brain barrier.

### 2.14 Quantitative real‐time PCR (qPCR)

Total RNA was extracted from tissues or cells using TRIzol reagent (Invitrogen, Carlsbad, CA, USA). The concentration and purity of the total RNA were subsequently measured. Reverse transcription was performed in accordance with the instructions of the Hifair® AdvanceFast 1st Strand cDNA Synthesis Kit (YEASEN, Shanghai, China). Next, the qPCR assay was conducted using Hieff® qPCR SYBR Green Master Mix (YEASEN). The relative expression levels of genes were computed via the 2^−ΔΔ*Ct*^ method. The primers were listed in Table 1.

**TABLE 1:** Sequences of primers for qPCR analysis.

Gene	Primer sequence
IL‐1*β*	Forward: 5′‐TGG ACC TTC CAG GAT GAG GAC A‐3′
	Reverse: 5′‐GTT CAT CTC GGA GCC TGT AGT G‐3′
IL‐6	Forward: 5′‐TAC CAC TTC ACA AGT CGG AGG C‐3′
	Reverse: 5′‐CTG CAA GTG CAT CAT CGT TGT TC‐3′
TNF‐*α*	Forward: 5′‐GGT GCC TAT GTC TCA GCC TCT T‐3′
	Reverse: 5′‐GCC ATA GAA CTG ATG AGA GGG AG‐3′
*β*‐actin	Forward: 5′‐CAT TGC TGA CAG GAT GCA GAA GG‐3′
	Reverse: 5′‐TGC TGG AAG GTG GAC AGT GAG G‐3′

### 2.15 ELISA

ELISA kits for IL‐1*β* (E‐EL‐M0037, Elabscience, Wuhan, China), IL‐6 (E‐EL‐M0044, Elabscience), and TNF‐*α* (E‐EL‐M3063, Elabscience) were used to detect their levels in tissues or cell supernatants.

### 2.16 Western blot

Total protein was extracted from brain tissues or cells using RIPA lysis buffer (Beyotime, Shanghai, China), and the protein concentration was assessed using a BCA kit (Beyotime). The protein sample (30 μg) was subsequently separated on an SDS–PAGE gel and transferred onto a PVDF membrane (Millipore). After being blocked with 5% nonfat milk, the membrane was incubated with the following primary antibodies: anti‐IL‐1*β* (ab283818, 1:1000, Abcam), anti‐TNF‐α (ab183218, 1:1000, Abcam), anti‐Bax (ab32503, 1:2000, Abcam), anti‐cleaved Caspase3 (#9661, 1:1000, Cell Signaling Technology, Danvers, MA, USA), anti‐Bcl‐2 (ab182858, 1:2000, Abcam), anti‐LC3B (ab48394, 1:1000, Abcam), anti‐p62 (ab109012, 1:10000, Abcam), anti‐PINK1 (23274‐1‐AP, 1:500, Proteintech), anti‐Parkin (14060‐1‐AP, 1:1000, Proteintech), anti‐*β*‐actin (20536‐1‐AP, 1:5000, Proteintech), and anti‐COX IV (11242‐1‐AP, 1:10000, Proteintech) at 4°C overnight. After washing, the membrane was exposed to the corresponding secondary antibodies for 1 h. Next, a BeyoECL Plus Kit (Beyotime) was used to visualize the imprinted bands, followed by a quantitative analysis using ImageJ software.

### 2.17 Flow cytometry assay of apoptosis

An Annexin V‐FITC Apoptosis Detection Kit (Beyotime) was used to assess apoptosis. Briefly, the cells (1 × 10^5^) were incubated in the dark with 5 μL of Annexin V‐FITC and 10 μL of propidium iodide for 20 min at 37°C. The percentage of apoptotic HT22 cells was subsequently determined via flow cytometry.

### 2.18 NeuN immunofluorescence staining and TUNEL staining

The brain tissue sections were fixed with paraformaldehyde (4%) and then blocked with bovine serum albumin (BSA; 5%) and Triton X‐100 (0.1%) for 1 h. Subsequently, the sections were incubated with an anti‐NeuN antibody (26975‐1‐AP, 1:100, Proteintech) at 4°C overnight, followed by an incubation with the secondary antibody at 37°C for 60 min. DAPI (Beyotime) was used for nuclear staining. After staining with the NeuN antibody, the sections were processed with a TUNEL Apoptosis Assay Kit (Beyotime). TUNEL‐positive neurons were observed under a fluorescence microscope for the evaluation of neuronal apoptosis.

### 2.19 The colocalization of TOM20 and LC3B

After treatment, the cells seeded on coverslips were fixed with 4% formaldehyde for 15 min and permeabilized with a solution containing 5% BSA and 0.1% Triton X‐100. Subsequently, the cells or tissue sections were incubated with primary antibodies against LC3B (ab48394, 1 μg/mL; Abcam) and translocase of the outer membrane 20 (TOM20; ab186735, 1:250; Abcam) overnight at 4°C. Next, secondary antibodies conjugated with Alexa 488 (1:200; ab150077, Abcam) or Alexa 594 (1:200; ab150080, Abcam) were incubated with the samples for 1 h in the dark at room temperature. The samples were counterstained with DAPI (Sigma‐Aldrich, USA) to visualize the nuclei. After mounting, images of TOM20 and LC3B colocalization were obtained under a confocal laser scanning microscope (Leica, Wetzlar, Germany) or a fluorescence microscope.

### 2.20 Statistical analysis

The data are presented as the means ± standard deviations and statistical analyses were conducted using GraphPad Prism 8.0.2 software (La Jolla, CA, USA). Comparisons between two groups were performed using Student's *t*‐test, whereas comparisons among multiple groups were performed using one‐way analysis of variance. A *p*‐value of less than 0.05 was regarded as statistically significant.

## 3 RESULTS

### 3.1 Identification of OM‐MSCs and exosomes

**FIGURE 1:**
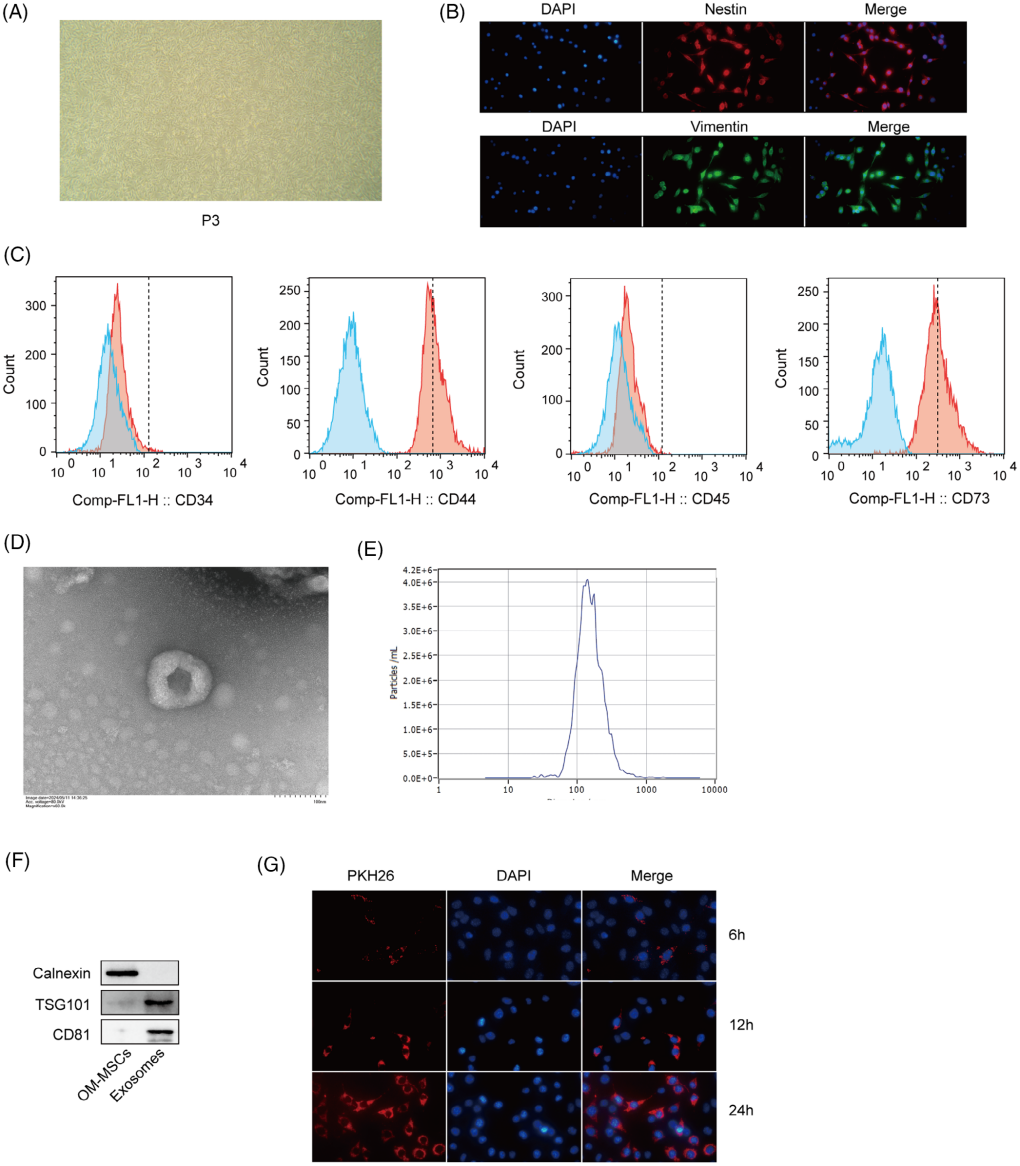
Identification of OM‐MSCs and exosomes. (A) Cell morphology was observed under a microscope. (B) Vimentin and Nestin levels were determined using immunofluorescence staining. (C) The expression of CD44, CD73, CD34, and CD45 was detected by flow cytometry. (D) Transmission electron microscopy was used to observe the morphology of the exosomes. (E) The peak particle size of the purified exosomes was assessed by NTA. (F) Western blot detection of CD81, TSG101, and Calnexin levels in purified exosomes. (G) Fluorescent PKH26‐labeled exosomes taken up by HT22 cells were observed via fluorescence microscopy. *n* = 3.

First, the OM‐MSCs were isolated and showed a typical spindle shape under a microscope (Figure 1A). The immunofluorescence results revealed that Vimentin and Nestin were expressed on the cell surface (Figure 1B). Moreover, positive expression of CD44 and CD73 and negative expression of CD34 and CD45 were detected by flow cytometry, indicating that the identified cells were OM‐MSCs (Figure 1C). Subsequently, exosomes derived from OM‐MSCs were extracted and characterized using transmission electron microscopy, revealing their spherical morphology. NTA revealed that the predominant particle size of the isolated exosomes was 125 nm, which aligns with the expected size range for exosomes (Figure 1D, E). Moreover, the purified exosomes expressed CD81 and TSG101, whereas Calnexin was not expressed (Figure 1F). The results of fluorescent PKH26 labeling indicated that HT22 cells were capable of internalizing exosomes derived from OM‐MSCs (Figure 1G).

### 3.2 OM‐MSC‐derived exosomes inhibit hemin‐induced inflammation and apoptosis in HT22 cells

**FIGURE 2:**
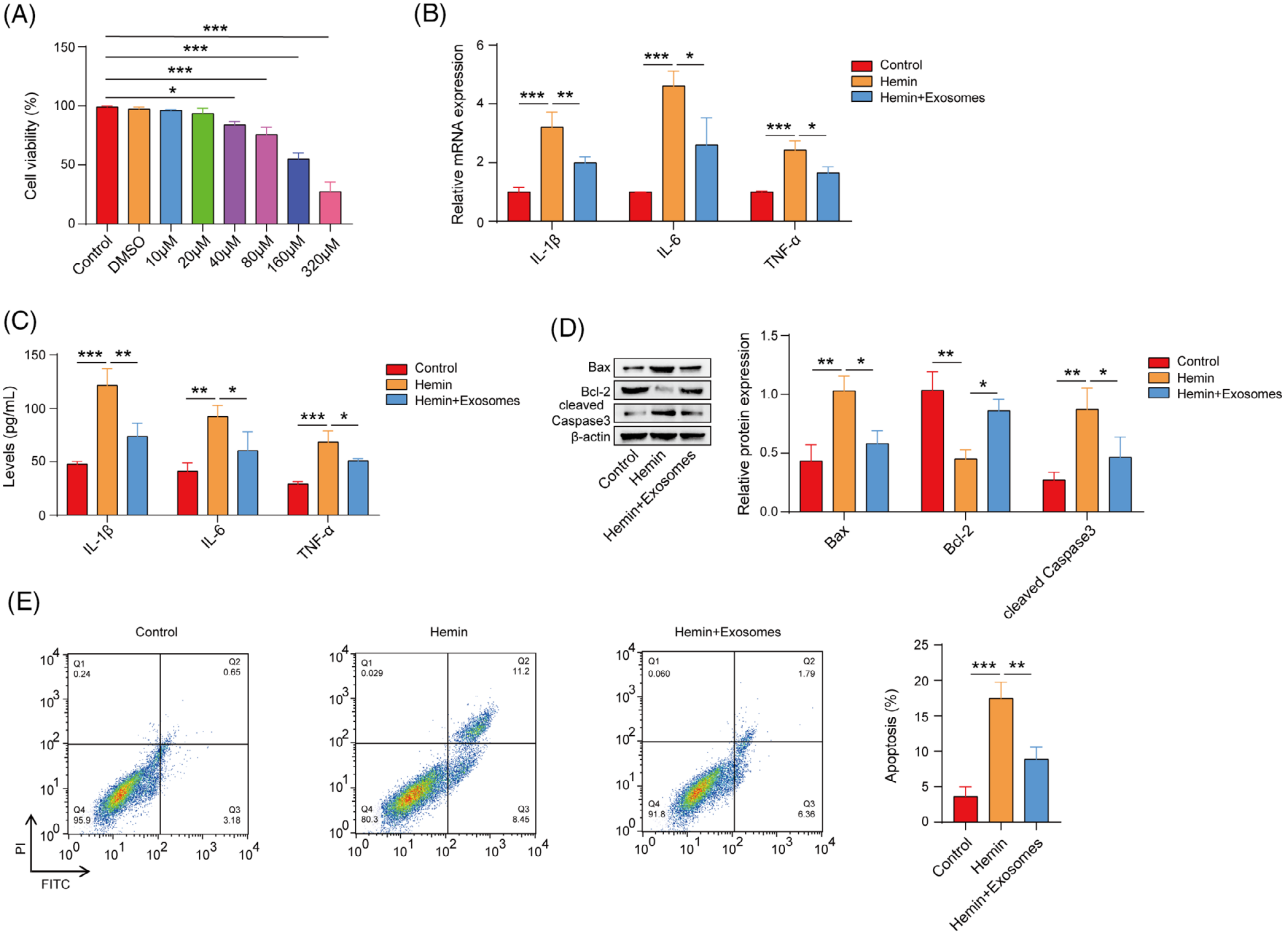
OM‐MSC‐derived exosomes inhibited hemin‐induced inflammation and apoptosis in HT22 cells. (A) A CCK‐8 assay was used to detect HT22 cell viability. (B) qPCR was performed to measure the mRNA expression levels of IL‐1*β*, IL‐6, and TNF‐*α*. (C) The levels of IL‐1*β*, IL‐6, and TNF‐*α* were detected using ELISAs. (D) Western blotting was performed to determine the levels of Bax, cleaved Caspase3, and Bcl‐2. (E) Flow cytometry was used to assess the degree of apoptosis in HT22 cells. *n* = 3. Hemin group versus control group; (hemin + exosome) group vs. hemin group. *p <0.05, **p <0.01, and ***p <0.001.

The results revealed that 40–320 μM hemin significantly reduced HT22 cell viability, and 160 μM hemin was selected to explore the effect of OM‐MSC‐derived exosomes (Figure 2A). The findings from qPCR and ELISAs indicated that hemin increased the mRNA expression and production of IL‐1*β*, IL‐6, and TNF‐*α*, changes that were partially mitigated by the administration of exosomes (Figure 2B, C). Furthermore, hemin increased the protein levels of Bax and cleaved Caspase3 but decreased the level of Bcl‐2. Exosomes significantly inhibited the regulatory effects of hemin on apoptosis‐related proteins (Figure 2D). Flow cytometry confirmed that the exosome treatment significantly inhibited the hemin‐induced apoptosis of HT22 cells (Figure 2E). Taken together, these findings suggest that exosomes derived from OM‐MSCs can inhibit neuroinflammation and neuronal apoptosis in vitro.

### 3.3 Exosomes derived from OM‐MSCs reduce neuroinflammation and neuronal apoptosis after SAH

**FIGURE 3:**
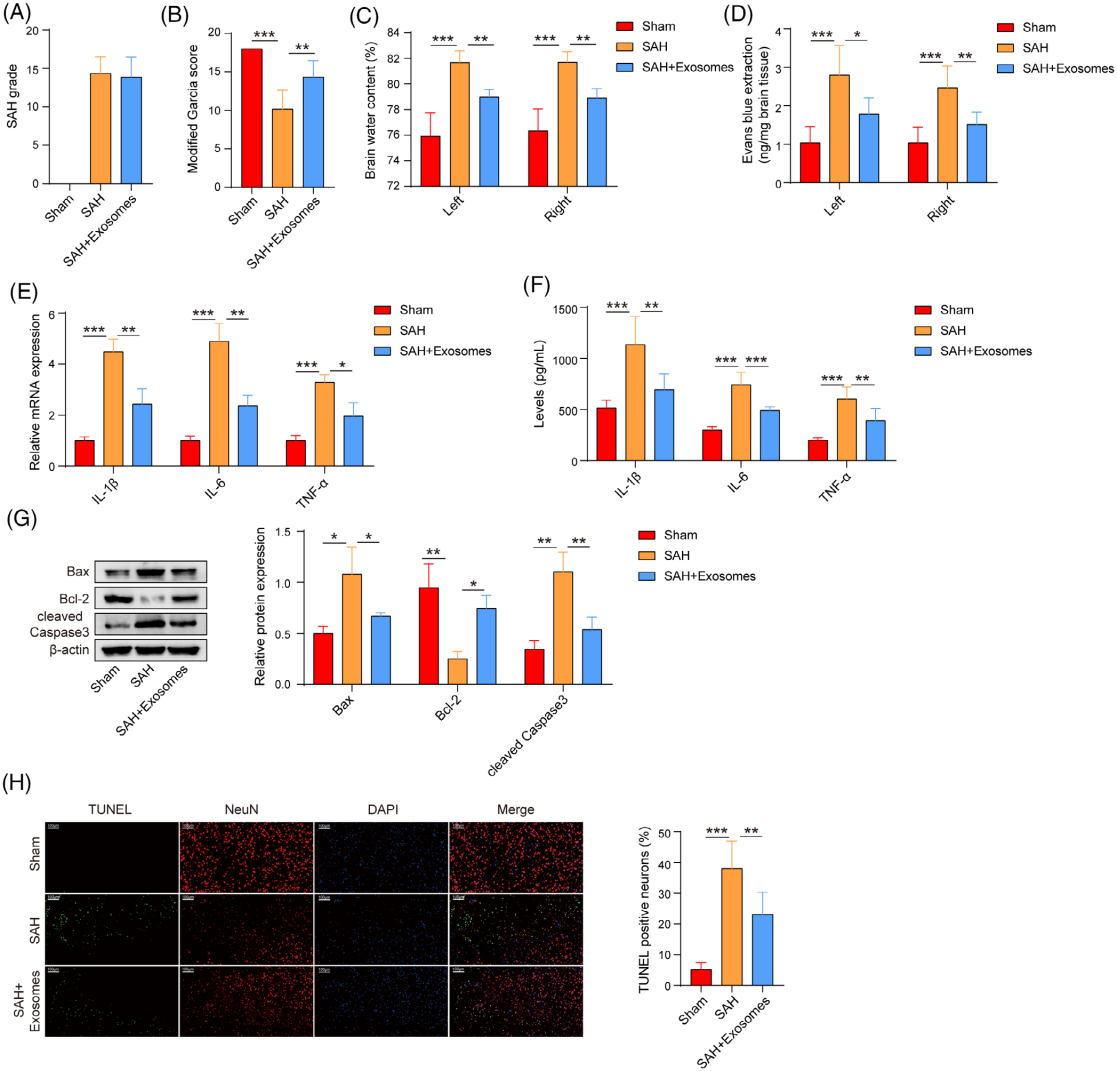
OM‐MSC‐derived exosomes reduce neuroinflammation and neuronal apoptosis after SAH. (A) SAH grades of the mice were assessed. (B) Modified Garcia scores were determined to assess neurological function. (C) Assessment of cerebral edema by measuring the brain water content in the mice. (D) Evans blue exudation experiments were performed to assess blood–brain barrier injury in the mice. *n* = 6. (E), (F) The expression of IL‐1*β*, IL‐6, and TNF‐*α* in the ipsilateral cortex of the mice was detected by qPCR (*n* = 3) and ELISAs (*n* = 6). (G) Western blot analysis of Bax, cleaved Caspase3, and Bcl‐2 levels in the ipsilateral cortex of the mice. *n* = 3. (H) NeuN immunofluorescence staining and TUNEL staining were performed to measure the degree of apoptosis of cortical neurons. *n* = 6. Sham group versus SAH group; SAH + exosomes group versus SAH group. *p <0.05, **p <0.01, and ***p <0.001.

Next, the effect of exosomes on neuronal damage following SAH in mice was confirmed. Mice whose SAH severity exceeded a score of 8 points were included in the study (Figure 3A). Neurological function was significantly impaired in SAH mice, as assessed by the modified Garcia score, whereas the exosome treatment improved the neurological function of SAH model mice (Figure 3B). Cerebral edema was observed in SAH mice, as assessed by the brain water content, and exosome treatment alleviated SAH‐induced cerebral edema (Figure 3C). Evans blue extravasation revealed that the exosome treatment significantly ameliorated blood–brain barrier damage in SAH mice (Figure 3D). The levels of IL‐1*β*, IL‐6, and TNF‐*α* that were upregulated by SAH were significantly reduced by exosome treatment in the ipsilateral cortex, as measured by qPCR and ELISAs (Figure 3E, F). In addition, the exosome treatment significantly decreased the levels of Bax and cleaved Caspase3 but increased the level of Bcl‐2 in the ipsilateral cortical tissue of SAH mice (Figure 3G). NeuN immunofluorescence staining and TUNEL staining indicated that exosomes inhibited the apoptosis of cerebral cortical neurons induced by SAH (Figure 3H). Our results suggested that OM‐MSC‐derived exosomes could reduce neuroinflammatory damage after SAH.

### 3.4 OM‐MSC‐derived exosomes inhibit hemin‐induced neuronal cell inflammation and apoptosis by activating mitophagy

**FIGURE 4:**
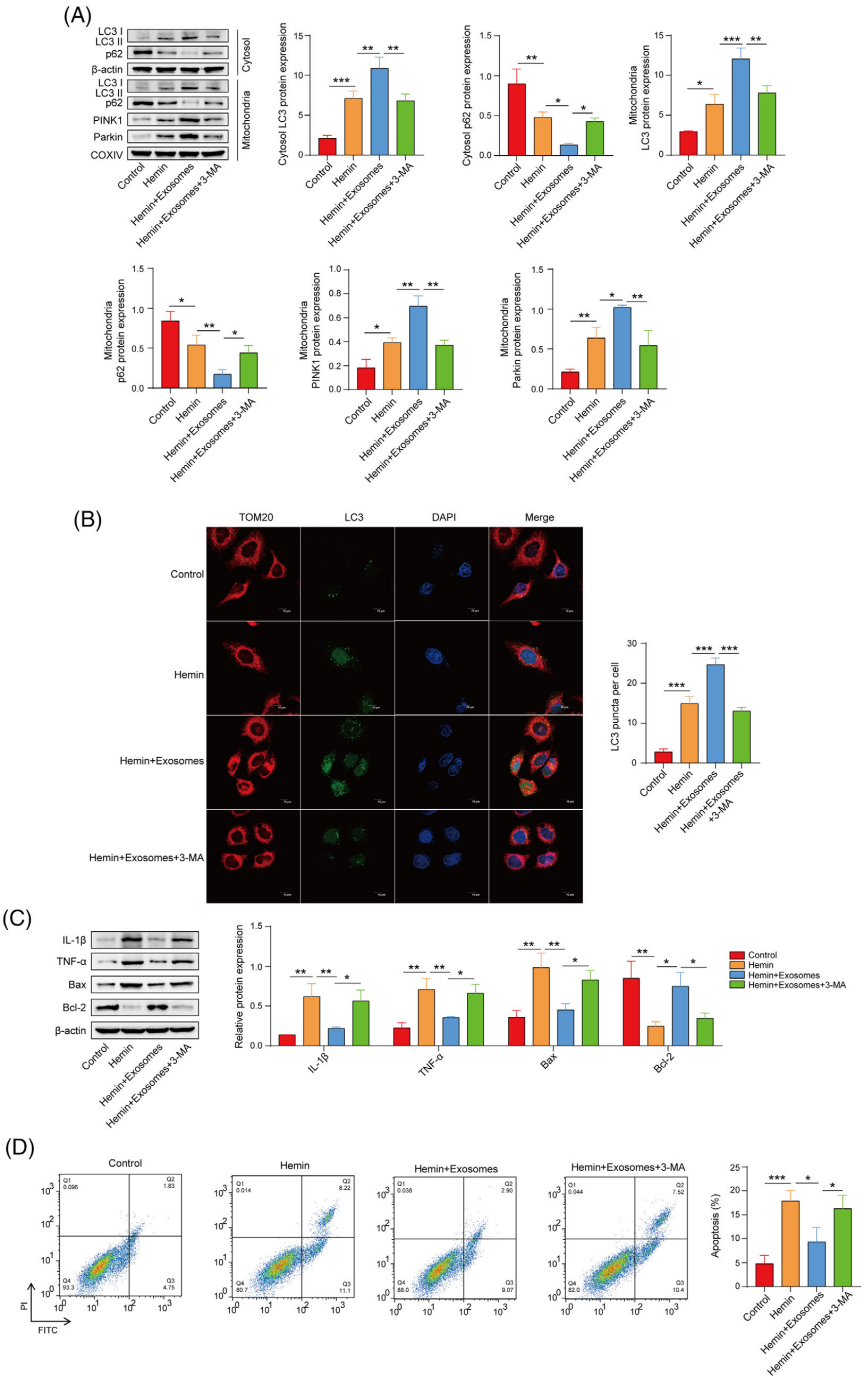
Exosomes derived from OM‐MSCs inhibit hemin‐induced neuronal inflammation and apoptosis by activating mitophagy. (A) Western blot analysis of cytoplasmic LC3B and p62, mitochondrial LC3B, PINK1 and Parkin levels. (B) Confocal microscopy detection of mitophagy (the colocalization of LC3B and TOM20) in HT22 cells. (C) Western blot analysis of IL‐1*β*, TNF‐*α*, Bax, and Bcl‐2 levels. (D) Flow cytometry was used to assess the degree of apoptosis in HT22 cells. *n* = 3. Hemin group versus control group; hemin + exosomes group versus hemin group; hemin + exosomes +3‐MA group versus hemin + exosomes group. *p <0.05, **p <0.01, and ***p<0.001.

Exosomes significantly increased the levels of cytoplasmic LC3B, mitochondrial LC3B, PINK1, and Parkin and decreased the cytoplasmic and mitochondrial p62 levels in hemin‐stimulated cells. However, the autophagy inhibitor 3‐MA partially reversed the effects of exosomes on the levels of mitophagy marker proteins (Figure 4A). The colocalization of LC3B and TOM20 was then detected. As shown in Figure 4B, exosomes increased the colocalization of LC3 and TOM20 in hemin‐stimulated cells, indicating increased mitophagy, which was inhibited by treatment with 3‐MA. Moreover, exosomes significantly decreased the levels of IL‐1*β*, TNF‐*α*, and Bax and increased the level of Bcl‐2. Nevertheless, the administration of 3‐MA partially counteracted the regulatory effects of exosomes on the modulation of inflammatory and apoptotic markers (Figure 4C). Flow cytometry revealed that the inhibition of mitophagy partially reversed the effect of exosomes on hemin‐induced apoptosis in HT22 cells (Figure 4D). These results indicate that exosomes derived from OM‐MSCs inhibit hemin‐induced inflammatory injury in neuronal cells by promoting mitophagy.

### 3.5 OM‐MSC‐derived exosomes reduce neuroinflammation and neuronal apoptosis after SAH by activating mitophagy

**FIGURE 5:**
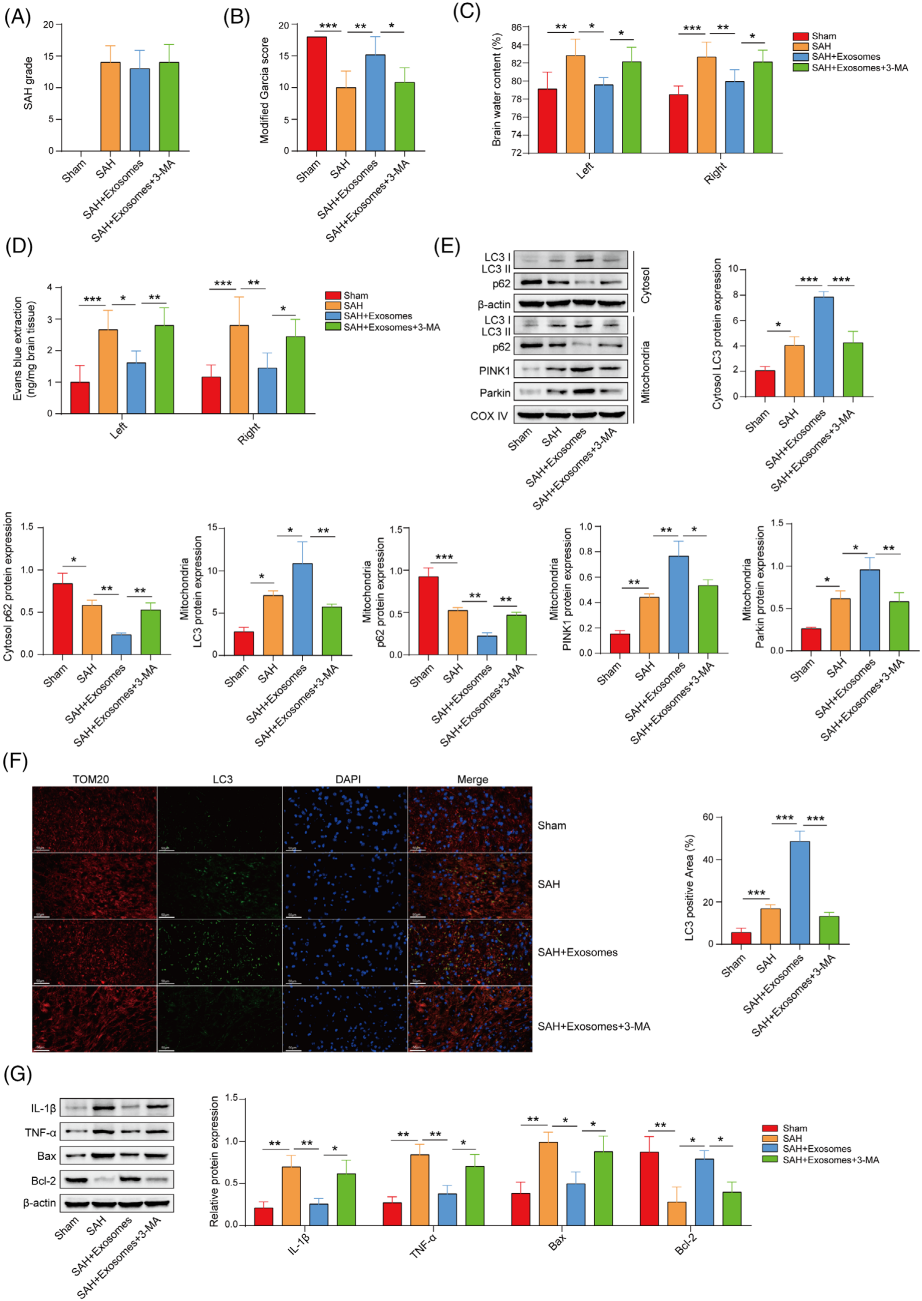
Exosomes derived from OM‐MSCs reduce neuroinflammation and neuronal apoptosis after SAH by activating mitophagy. (A) SAH grades. (B) Modified Garcia scores of the mice. (C) Evaluation of the brain water content in the mice. (D) The Evans blue exudation test was used to assess blood–brain barrier injury in mice. *n* = 6. (E) Western blot analysis of cytoplasmic LC3B and p62 levels and mitochondrial LC3B, PINK1 and Parkin levels in the cerebral cortex of mice. *n* = 3. (F) Immunofluorescence detection of mitophagy (the colocalization of LC3B and TOM20) in the cerebral cortex of mice. *n* = 6. (G) Western blot analysis of the levels of IL‐1*β*, TNF‐*α*, Bax, and Bcl‐2. *n* = 3. Sham group versus SAH group; SAH + exosomes group versus SAH group; SAH + exosomes +3‐MA group versus SAH + exosomes group. *p <0.05, **p <0.01, and ***p <0.001.

The SAH grade and modified Garcia scores revealed that 3‐MA suppressed the improvement in the neurofunction of SAH mice induced by the exosomes (Figure 5A, B). The evaluation of the brain water content suggested that 3‐MA significantly inhibited the ability of exosomes to reduce brain edema in SAH mice (Figure 5C). The results of the Evans blue exudation test indicated that 3‐MA significantly inhibited the protective effect of exosomes on blood–brain barrier injury in SAH mice (Figure 5D). Western blot analysis revealed that exosomes significantly increased the levels of LC3B, PINK1, and Parkin in mitochondria and decreased the level of p62 in SAH mice. 3‐MA partially reversed the regulatory effects of exosomes on the levels of mitophagy‐associated proteins (Figure 5E). Immunofluorescence images revealed that exosomes promoted the colocalization of LC3B and TOM20 in SAH mice; however, 3‐MA suppressed their colocalization (Figure 5F). Additionally, exosomes significantly decreased the levels of IL‐1*β*, TNF‐α, and Bax and increased the level of Bcl‐2, and 3‐MA partially reversed the effects of exosomes on the levels of markers of inflammation and apoptosis in SAH mice (Figure 5G). Overall, OM‐MSC‐derived exosomes alleviate neuronal inflammatory damage after SAH by promoting mitophagy.

## 4 DISCUSSION

SAH, a complex type of cerebrovascular disease, accounts for 5%–10% of all strokes and occurs in people aged 40–60 years.^7^ Approximately 35% of survivors suffer permanent disability, cognitive impairment (especially reduced executive ability and short‐term memory), and mental health symptoms (such as anxiety and depression) after SAH, which can lead to a significant decline in quality of life.^25^ EBI is a key factor that causes delayed cerebral ischemia and neurological dysfunction in patients with SAH, and the early regulation of oxidative stress and the neuroinflammatory response is an effective measure to improve the prognosis of neurological function in patients with SAH.^25^, ^26^ In this study, we observed that exosomes derived from OM‐MSCs alleviate EBI after SAH and hemin‐induced inflammatory damage to neuronal cells by activating mitophagy, suggesting the potential of OM‐MSC‐derived exosomes as a novel therapeutic approach.

Neuroinflammation plays a key role in EBI following SAH by promoting neuronal apoptosis, activating astrocytes to destroy the blood–brain barrier, leading to brain edema, and recruiting peripheral neutrophils to the intracranial microvascular system, leading to vasodilation disorders, microthrombosis, cerebral hypoperfusion and other mechanisms.^27^ Early administration of anti‐inflammatory or anticytokine therapy after SAH can restrain EBI by inhibiting neuroinflammation to improve the prognosis of SAH patients.^28^ IL‐1 receptor antagonists play a protective role by blocking the heme‐driven inflammatory response after SAH in patients.^29^ Atorvastatin can reduce EBI after SAH in mice by inhibiting the inflammatory response through the pyroptosis pathway.^30^ Our study revealed that exosomes derived from OM‐MSCs can inhibit neuroinflammation after SAH, thus exerting a significant therapeutic effect.

OM‐MSCs are a type of stem cell that was first discovered in the human olfactory mucosa by Huard et al. in 2010 and their potential effects on the treatment of cerebral vascular‐related disorders have received increasing attention.^31^ Following cerebral ischemia/reperfusion injury, OM‐MSCs can secrete neuroprotective factors to alleviate the Golgi stress response.^17^ In addition, OM‐MSC coculture improved neuronal injury after treatment with hemin, and OM‐MSC intracerebral transplantation inhibited intracerebral hemorrhage‐induced neuronal death.^32^ OM‐MSCs are ideal cells for injury repair, but little is known about the functional roles of OM‐MSC‐derived exosomes. Exosomes derived from OM‐MSCs may have a potential function in neurorestorative treatment.^22^ OM‐MSC‐derived exosomes can modulate glial cell activation, diminish neuroinflammation, and promote brain‐derived neurotrophic factor‐associated synaptic plasticity and neurogenesis.^18^ In this study, we used a variety of experimental methods to identify OM‐MSCs and isolated exosomes. We also found that OM‐MSC‐derived exosomes inhibited hemin‐induced neuronal inflammation and alleviated neuroinflammatory damage after SAH.

EBI is a very complex pathological process occurring after SAH that involves increased intracranial pressure, cerebral microvascular dysfunction, blood–brain barrier breakdown, nerve cell apoptosis, oxidative stress, and mitochondrial dysfunction. Among them, mitophagy is an important process used by cells to maintain self‐homeostasis. Mitophagy is closely related to EBI after SAH, and neuroinflammation can be inhibited by activating mitophagy.^33^ Triiodothyronine inhibits microglial activation and neuronal inflammation and reduces neuronal apoptosis following SAH by promoting mitophagy.^34^ Furthermore, the NLRP3 inflammasome is activated in the early stage after SAH, resulting in inflammatory responses. The inhibition of the mitophagy‐associated NLRP3 inflammasome might provide neuroprotection against EBI after SAH.^20^ Our experimental results showed that OM‐MSC‐derived exosomes alleviated inflammation and neuronal injury after SAH by activating mitophagy in vitro and in vivo. Exosomes play crucial roles in intercellular communication by delivering RNAs (including mRNAs, microRNAs, lncRNAs, or circRNAs) and proteins to recipient cells.

However, this study has several limitations. First, the specific molecular mechanisms of exosome uptake and function in recipient cells remain unclear. We explored mitophagy but overlooked other potential pathways and mediators. Exosomes carry diverse RNAs and proteins, yet we did not comprehensively analyze which ones modulate neuronal damage. Future studies can address this limitation by screening key molecules using RNA sequencing and proteomics, facilitating a deeper understanding and targeted therapy development. Second, neurological performance was evaluated 24 h after SAH infection, which is associated with SAH‐induced EBI; however, the extent of secondary brain injury is also worth evaluating to confirm the final outcome of SAH. Third, the in vivo study was only conducted in a mouse model, which differs from human SAH pathophysiology. Mice have distinct brain structures, immune responses, and metabolic rates. For better clinical translation, larger animal models such as pigs or nonhuman primates, which are closer to humans anatomically and physiologically, could be considered. Additionally, multicenter clinical trials are needed to evaluate exosome safety and efficacy in SAH patients, strengthening the clinical evidence. Finally, our experimental design focused on a specific time window for assessing the effects of exosomes. However, SAH pathology is dynamic; the optimal treatment time or duration may vary among patients and disease stages. Future experiments should explore broader time points and regimens. Longitudinal studies can monitor changes over time to refine the clinical application protocol and maximize exosome benefits.

## 5 CONCLUSIONS

Our study verified that exosomes derived from OM‐MSCs exert significant regulatory effects on neuroinflammation and neuronal apoptosis following SAH. Exosomes from OM‐MSCs suppressed the hemin‐induced inflammation and apoptosis of neuronal cells by activating mitophagy and mitigated neuroinflammation and neuronal injury after SAH. Hence, these discoveries provide a reference for the clinical presentation of neuroinflammation after SAH and experimental data for the development of novel technologies and targeted drugs.

## CONFLICT OF INTEREST STATEMENT

The authors declare that they have no known competing financial interests or personal relationships that could have appeared to influence the work reported in this paper.

## Data Availability

The data that support the findings of this study are available from the corresponding author upon reasonable request.
